# Genome Editing and Cardiac Arrhythmias

**DOI:** 10.3390/cells12101363

**Published:** 2023-05-11

**Authors:** Oliver M. Moore, Kevin S. Ho, Juwan S. Copeland, Vaidya Parthasarathy, Xander H. T. Wehrens

**Affiliations:** 1 Cardiovascular Research Institute, Baylor College of Medicine, Houston, TX 77030, USA; 2 Department of Integrative Physiology, Baylor College of Medicine, Houston, TX 77030, USA; 3 Department of Neuroscience, Baylor College of Medicine, Houston, TX 77030, USA; 4 Department of Molecular and Human Genetics, Baylor College of Medicine, Houston, TX 77030, USA; 5 Department of Medicine, Baylor College of Medicine, Houston, TX 77030, USA; 6 Department of Pediatrics, Baylor College of Medicine, Houston, TX 77030, USA; 7 Center for Space Medicine, Baylor College of Medicine, Houston, TX 77030, USA

**Keywords:** arrhythmias, catecholaminergic polymorphic ventricular tachycardia, CRISPR/Cas9, genome editing, long QT syndrome, ryanodine receptor, RyR2

## Abstract

This article reviews progress in the field of cardiac genome editing, in particular, its potential utility in treating cardiac arrhythmias. First, we discuss genome editing methods by which DNA can be disrupted, inserted, deleted, or corrected in cardiomyocytes. Second, we provide an overview of in vivo genome editing in preclinical models of heritable and acquired arrhythmias. Third, we discuss recent advancements in cardiac gene transfer, including delivery methods, gene expression optimization, and potential adverse effects associated with therapeutic somatic genome editing. While genome editing for cardiac arrhythmias is still in its infancy, this approach holds great promise, especially for inherited arrhythmia syndromes with a defined genetic defect.

## 1. Introduction

Millions of people around the world experience cardiac arrhythmias at some point during their life [1]. While arrhythmias are harmless most of the time, some heart rhythm disorders can have serious consequences or may result in death. The most common arrhythmia type is atrial fibrillation, which can lead to life-threatening complications such as stroke or heart failure [2]. Though less common, ventricular arrhythmias such as ventricular tachycardia and ventricular fibrillation can be very dangerous and lead to sudden cardiac death [3,4]. 

Despite advances in screening and preventative treatments, the estimated lifetime risk for premature death due to arrhythmogenic sudden cardiac death remains high (approximately 1 in 9 men and 1 in 30 women) [5]. In part, this can be explained by risk factors including structural heart disease, coronary artery disease, nutrition, and genetic predispositions [6]. While acquired co-morbidities such as coronary artery disease are leading causes of sudden cardiac arrest in older adults, arrhythmias resulting from heritable diseases are the leading causes of sudden cardiac arrest in children and young adults [3]. 

A growing understanding of the genetics underlying cardiac arrhythmias has enabled new treatment possibilities including the use of cardiac genome editing [7]. Monogenetic diseases associated with cardiac arrhythmias include congenital long QT syndrome (LQTS), short QT interval syndrome, Brugada syndrome, catecholaminergic polymorphic ventricular tachycardia (CPVT), idiopathic ventricular fibrillation, Wolff–Parkinson–White syndrome, arrhythmogenic cardiomyopathy, and other cardiomyopathy syndromes associated with an increased risk of arrhythmias such as dilated cardiomyopathy (DCM), hypertrophic cardiomyopathy (HCM) and hereditary transthyretin amyloidosis (ATTR-CM) [8]. Acquired arrhythmias such as atrial fibrillation or those associated with ischemic heart disease are probably harder to treat using genome editing, although targeting a gene that is a master regulator in the pathogenesis might be feasible. 

Advances in cardiac gene therapy and viral delivery methods have improved the efficacy of somatic genome editing in the heart [9]. Although other forms of cardiac gene therapy are limited by difficulties in controlling expression levels and potential compensatory mechanisms [10], cardiac genome editing is more precise, permanent, and can directly address the underlying disease-causing mechanisms. Recent studies have demonstrated successful cardiac gene editing in several preclinical models as effective treatments for arrhythmogenic cardiomyopathies and hereditary arrhythmia syndromes [11]. However, in contrast to other types of gene therapy, cardiac gene editing has its own unique challenges that have delayed its use in clinical trials. In this review, we will cover relevant genome editing techniques by which DNA can be disrupted, inserted, deleted, or corrected in cardiomyocytes, review in vivo genome editing in preclinical models of heritable and acquired arrhythmias, and discuss current limitations and future perspectives on the clinical impact of cardiac genome editing. 

## 2. Genome Editing Techniques

While gene targeting in embryonic cells has been used for modeling familial cardiac disorders, somatic cardiac genome editing only became feasible following the development of nucleases that can introduce double-stranded breaks (DSBs) at specific locations in the genome. These nucleases can be categorized into four general categories: meganucleases, zinc-finger nucleases (ZFN), transcription activator-like effector nucleases (TALENs), and clustered regularly interspaced short palindromic repeats (CRISPR) associated with nuclease 9 (Cas9) [12]. Notably, however, only the CRISPR/Cas9 system has been used for therapeutic somatic genome editing of the heart. Therefore, we will focus mainly on the CRISPR/Cas9 system for the remainder of this review. 

**Figure 1:**
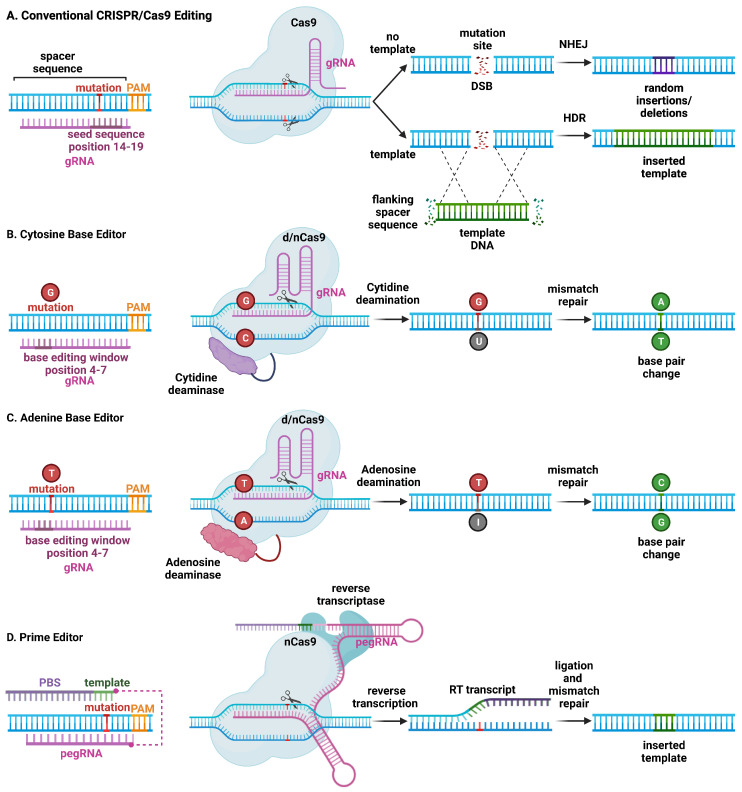
Schematic overview of major CRISPR/Cas9-based genome editing technologies. (**A**) Conventional CRISPR/Cas9 editing of a mutant allele involves selecting guide RNAs adjacent to a protospacer adjacent motif (PAM) site, with a mutation in the spacer sequence (14–19) to minimize wild-type allele cleavage. The Cas9 endonuclease introduces a double-stranded break (DSB). Non-homologous end joining (NHEJ) repairs the mutant allele but introduces insertions and deletions at the mutation site, which can lead to frame-shift mutations and stop codons, resulting in nonsense-mediated decay of mutant RNA (top). On the other hand, homology-directed repair (HDR) is less efficient but leads to correction of the mutant allele using a DNA repair template. (**B**) Cytosine base editors are created by fusing Cas9 nickase (nCas9) or catalytically inactive “dead” Cas9 (dCas9) to a cytidine deaminase. Base editors are targeted to a specific locus using gRNA. They can convert cytidine (C) to uridine (U) within a small editing window (4–7 in the spacer sequence, depending on the editor type), near the PAM site. Uridine is subsequently converted to thymidine (T) through base excision repair. (**C**) Likewise, adenosine base editors have been engineered to convert adenosine (A) to inosine (I), which is subsequently converted to guanidine (G). (**D**) Prime editing involves a reverse transcriptase and dCas9 that can produce single-stranded DNA breaks. The 3′-extended single guide RNA contains a primer binding site and a reverse transcriptase template, which is referred to as primer editing guide RNA (pegRNA). Hybridization of the exposed 3′-end to the primer binding site primes the reverse transcription of the nicked DNA strand for the desired edit.

In the CRISPR/Cas9 system, a synthetic RNA spacer sequence is inserted into a scaffold for the Cas9 nuclease [13,14]. This guide RNA (gRNA) sequence allows Cas9 to target genomic sequences of 20 or more base pairs (bp). Cas9 binds DNA at the protospacer adjacent motif (PAM) and cleaves 3 bp upstream of the PAM (Figure 1). In contrast to the amino acid recognition sequences that other endonucleases rely on, the CRISPR/Cas9 spacer sequences are much simpler to design, synthesize, and validate. As such, the field of genome editing overall has largely moved to this platform, and subsequent discoveries in cardiac gene editing have relied on utilizing the CRISPR/Cas9 system. The first publication of somatic genome editing of the heart involved using this system to correct Duchenne’s muscular dystrophy (DMD) in a mouse model [15]. Subsequently, there have been several developments in expanding the capabilities and efficacy of the CRISPR/Cas9 systems and these are reviewed in the subsequent sections [16].

The expression of CRISPR/Cas9 with a single gRNA is sufficient to create a DSB at a target genome locus. In cardiomyocytes, this has been shown to cause primarily non-homologous end-joining (NHEJ) repair (Figure 1) [17]. The lack of fidelity in NHEJ repair, however, results in insertions and deletions (indels) that can result in a frame shift, which—when introduced within a coding sequence—may lead to nonsense-mediated degradation. This method has been employed to model loss-of-function mutations by disrupting a target gene. In addition, this method can be used to disable a toxic or detrimental gain-of-function allele in heterozygous individuals. The feasibility of postnatal gene editing was demonstrated in mice overexpressing a cardiomyocyte-specific Cas9 in combination with adeno-associated viral (AAV) vector-mediated delivery of guide RNAs targeting *Myh6*, *Sav1*, and *Tbx20* [18]. Following AAV delivery, mosaic disruption of the target genes was seen, and only disruption of *Myh6* was sufficient to induce a cardiac phenotype. Despite the limitations of a mosaic knockdown in terms of efficiency, this method has been uniquely beneficial for characterizing a novel loss-of-function arrhythmia disorder that would otherwise be lethal in a complete knockout state [19]. NHEJ editing has also been effective in deleting splice donor or acceptor site sequences in out-of-frame exons, thereby restoring the open reading frame of the gene of interest. Several groups have demonstrated the restoration of cardiac dystrophin levels using AAV-mediated delivery of Cas9 and a single gRNA in mouse models of Duchenne muscular dystrophy DMD [20,21,22,23].

CRISPR/Cas9 genome editing can also be used to replace and correct the target sequence with a template through homology-directed repair (HDR) (Figure 1). Although gene correction is attained, HDR is less efficient compared with NHEJ, and a vast majority of edits will contain random insertions or deletions. Given the low efficiency in non-dividing cells such as cardiomyocytes, HDR has not been used (yet) for the treatment of cardiac arrhythmias in preclinical animal models. Another CRISPR technique involves using at least one gRNA sequence and a template sequence flanked by the gRNA target sequences in reverse orientation to create a DSB at the target site, followed by insertion of a corrected gene by incorporation of the template DNA through homology-independent targeted integration (HITI) [24]. Due to the limited efficacy of HDR in post mitotic tissue, HITI has not been used to correct arrhythmia phenotypes, although it was used to increase full-length dystrophin expression in a humanized mouse model of DMD with a genomic correction rate of 4–7% in cardiomyocytes [25].

Alternative CRISPR/Cas9-based genome editing techniques have been developed that allow for the introduction of point mutations in the genomic DNA without generating DSBs [26]. By fusing Cas9 nickase (nCas9) or deactivated Cas9 (dCas9) to a deaminase enzyme, the resulting base editor can edit DNA without DSBs, converting C to T, or G to A. Further development of cytosine base editors (CBE) and adenine base editors (ABE) resulted in expansions of possible conversions including C to T, A to G, T to C, and G to A, respectively [26]. Studies revealed an in vitro efficiency of 15–75%, with less than 1% indel formation, although efficiency is much lower in non-dividing cells. Moreover, DNA base editors have some shortcomings, including off-target DNA editing, the generation of bystander mutations, and promiscuous deamination effects in both DNA and RNA. Finally, prime editing uses a fusion protein, consisting of a catalytically impaired Cas9 endonuclease fused to an engineered reverse transcriptase, and a prime editing guide RNA (pegRNA), capable of replacing the target DNA nucleotides without the need for DSBs or donor DNA templates [27]. 

## 3. Using CRISPR/Cas9 Genome Editing to Create Animal and Cellular Arrhythmia Models

The advent of genome editing approaches such as CRISPR/Cas9 has created a whole new set of possibilities to generate animal models for biomedical research using virtually any species. Animal models in which human gene variants are introduced can provide convincing evidence for a disease-causing pathogenic mechanism [28]. While gene overexpression or knockdown strategies may provide useful insights in some cases [29], knock-in strategies that involve introducing the exact genetic variant in the model system are generally superior [30]. The RNA-guided Cas9 nuclease system can be used to easily generate knock-in mouse models [31]. This system has superseded all other systems for genome editing because of its simplicity of use, lower costs, and higher efficiency [32]. These days, most researchers prefer this approach over ES cell-based gene-targeting methods [33]. The CRISPR/Cas9 system allows for the introduction of footprint-free point mutations on various genetic backgrounds. While many of the initial mouse models of monogenetic arrhythmia syndromes were generated using ES cell-based methods, several newer models have been generated using CRISPR/Cas9 gene editing [34,35].

Genome editing can also be used to introduce potential disease-causing variants in patient-derived induced pluripotent stem cells (iPSCs) that can subsequently be differentiated into cardiomyocytes [36]. This approach has been particularly helpful to elucidate whether genetic variants of unknown significance (VUSs) in various genes purported to cause arrhythmias are in fact pathogenic [37]. Various genome editing techniques including CRISPR/Cas9 can introduce or correct genetic variants in iPSC before differentiation into cardiomyocytes, providing high throughput empirical evidence for characterizing novel mutations, their mechanisms, and potential therapies [38]. Many iPSC models of heritable arrhythmias, including LQTS, Brugada syndrome, CPVT, and arrhythmogenic cardiomyopathy, have been generated, as recently reviewed by Yang et al. [39]. These models have been used not only to characterize disease mechanisms but also to test the effectiveness of novel therapeutics [40].

In addition to being a tool for introducing genetic variants into iPSCs, CRISPR/Cas9 also enables efficient correction of inherited variants in iPSCs. By taking somatic cells such as skin fibroblasts or peripheral whole blood samples from patients, patient-derived iPSCs can be generated that include all VUSs present in the patient genome. Generating an isogenic control provides incredibly powerful evidence on whether a specific SNP is likely to be pathogenic. Most studies to this effect have used HDR to generate isogenic controls [41]. However, recent studies have begun to use more advanced techniques such as base editing and prime editing to correct specific variants in iPSCs [42]. The ease with which iPSCs can undergo genome editing has dramatically increased the number of VUS that can be characterized and has also expanded the genetic approaches that can be used to explore disease mechanisms. There are important limitations to the use of iPSC-derived cardiomyocytes (iPSC-CMs). While studies in single iPSC-CMs might yield new insights about certain inherited arrhythmia syndromes, it would be implausible that relevant insights could be gleaned from iPSC-CMs generated from patients with atrial fibrillation or ischemic heart disease. In addition, despite improvements in experimental protocols, iPSC-CMs still exhibit relatively immature and variable phenotypes. Finally, iPSC-CMs often do not have a functional SR, and the intracellular Ca^2+^ handling dynamics are quite different from those in freshly isolated CMs from adult animals or human patients [43].

## 4. Therapeutic Genome Editing in Preclinical Arrhythmia Models

**Figure 2:**
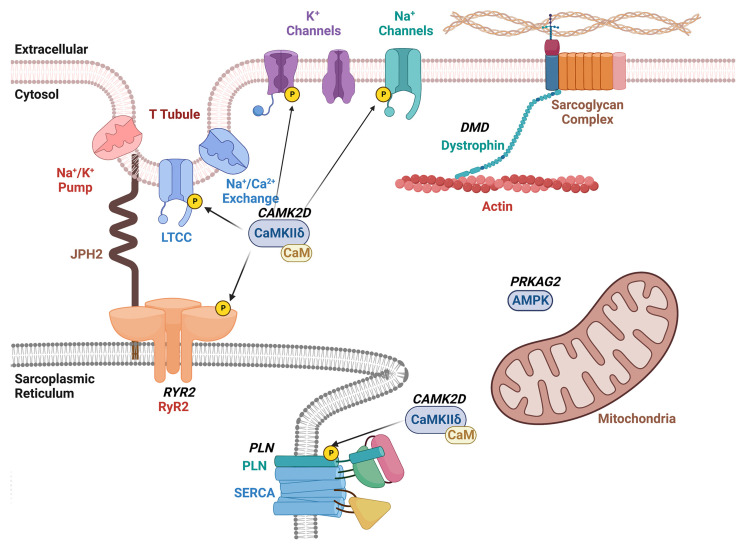
Schematic overview of therapeutic CRISPR/Cas9 targets for arrhythmia treatment. The diagram shows the plasma membrane with various ion channels including the voltage-gated L-type Ca^2+^ channel (LTCC), and K^+^ and Na^+^ channels. The sarcoplasmic reticulum (SR) is shown with the ryanodine receptor type-2 (RyR2)/Ca^2+^ release channel and sarco-/endoplasmic Ca^2+^-ATPase (SERCA2a) with its regulatory subunit phospholamban (PLN). Dystrophin links the plasmalemmal sarcoglycan complex to the sarcomere. The Ca^2+^/calmodulin-dependent protein kinase II (CaMKII) phosphorylates various ion channels and Ca^2+^ handling proteins within cardiomyocytes. Gene names that are targets of therapeutic genome editing are indicated in bold and italicized.

Somatic genome editing has also been used to correct heart disease in preclinical model systems (Table 1, Figure 2). For example, the CRISPR/Cas9 editing method was employed in correcting the cardiac *PRKAG2* syndrome, which is known to cause familial Wolff–Parkinson–White syndrome with cardiomyopathy [44]. *PRKAG2* syndrome is an autosomal-dominant inherited disease caused by missense mutations in the *PRKAG2* gene, which encodes the γ2 regulatory subunit of AMP-activated protein kinase [45]. Altered activity of this AMP-activated protein kinase results in excessive cellular glycogen deposition leading to cardiomyopathy and supraventricular arrhythmias. Systemic administration of adeno-associated virus serotype 9 (AAV9) with gRNA and the CRISPR/spCas9 gene-editing system was sufficient to disrupt the mutant *PRKAG2* allele in mice while leaving the wild-type allele intact [46]. While the genome-editing efficiency was relatively low (∼6.5% in mice injected at postnatal day (P) 4 and ∼2.6% in mice injected at P42), this treatment strategy reduced preexcitation arrhythmias by 40%, and restored the morphology and function of the heart in mutant mice [46].

Our research group utilized a similar approach to treat catecholaminergic polymorphic ventricular tachycardia (CPVT) in a preclinical mouse model [47]. CPVT is an autosomal-dominant inherited disease caused primarily by missense mutations in the *RYR2* gene that encodes the ryanodine receptor type 2 (RyR2) intracellular calcium channel [49]. The mutated channel generates a diastolic calcium leak that can trigger lethal arrhythmias [30]. We found that systemic administration of AAV to deliver gRNA and SaCas9 was sufficient to disrupt the mutant *RyR2* allele in a heterozygous mice model while leaving the wild-type allele intact. The editing frequency based on next generation sequencing was found to be around 11% in these *RyR2* mutant mice [47]. In addition, the reduced mutant allele mRNA levels were indicative of nonsense-mediated decay. This degree of genome editing reduced the ventricular tachycardia incidence by 100% and normalized channel function at the cellular level [47].

The same NHEJ pathway was induced by CRISPR/Cas9 genome editing in a humanized mouse model of arrhythmogenic dilated cardiomyopathy caused by a truncating variant in phospholamban (PLN) [48]. The PLN-R14del mutation was first discovered in a large Greek family with clinical signs of both dilated cardiomyopathy (DCM) and arrhythmogenic cardiomyopathy (ACM) [50]. Subsequently, many PLN-R14del mutation carriers were found in the Netherlands, where the original mutation is believed to have originated [51]. Mice harboring the R14del mutant human PLN were found to be more susceptible to rapid pacing-induced ventricular tachycardia. In vivo gene editing using AAV9 led to a greater than two-fold reduction in pacing-induced VT incidence and significantly raised the frequency threshold for induction [48]. 

While gene disruption of dominant-negative mutations is one of the most efficient methods of therapy, other methods of genome editing are needed in the case of haplo-insufficiency. Duchenne muscular dystrophy (DMD) is a fatal X-linked recessive disorder that causes progressive neuromuscular weakness and wasting. A quarter of DMD patients die from cardiac causes, and half of these deaths are due to lethal ventricular tachycardia [52]. Abnormal Ca^2+^ release from the sarcoplasmic reticulum via RyR2 overactivated by Ca^2+^/calmodulin-dependent protein kinase II (CaMKII) is a major mechanism underlying arrhythmogenesis in mice with DMD [53]. Inherited variants in the *DMD* gene usually involve single or multi-exon deletions that disrupt the open reading frame (ORF) and introduce a premature stop codon, resulting in a nonfunctional, truncate dystrophin protein [54]. CRISPR/Cas9 has been used to generate indels using NHEJ in the *DMD* gene to restore the ORF in rodent and large animal preclinical models [20]. Recently, Chemello et al. [42] used an adenine base editor (ABE) delivered using AAV9 as a split-intein system to restore protein expression in a DMD mouse model. By means of a single-swap base pair transition in the dinucleotide splicing motif, exon skipping was accomplished with restoration of dystrophin levels. The on-target editing efficiency ranged from 6.7 to 35.0%, with no notable editing at off-target sites [42]. Whereas the effects of base editing on arrhythmogenesis were not assessed in vivo, complementary studies in human induced-pluripotent stem cell-derived cardiomyocytes revealed that base editing normalized arrhythmic calcium handling deficits [42]. Additional studies are needed to determine whether base editing can also block lethal arrhythmias in vivo in animal models of DMD.

Finally, it may be possible to use genome editing for the treatment on non-genetic arrhythmia disorders by targeting key molecular switches that drive disease development. For example, Lebek et al. [55] recently showed that base editing could be used to eliminate oxidation-sensitive methionine residues on CaMKII [56] to confer protection from ischemia/reperfusion injury, which is often associated with ventricular arrhythmias. Since the same CaMKII residues have been implicated in ventricular arrhythmogenesis in DMD [53] and atrial fibrillation [57], similar approaches may hold promise for a wider array of arrhythmia disorders.

## 5. Current Challenges of Genome Editing in Arrhythmias and Future Developments

While CRISPR Cas9 genome editing can provide long-term correction to arrhythmia disorders, one major disadvantage of using CRISPR/Cas9 genome editing is that side effects can also be long-lasting. As such, an important question that remains is what amount of correction is needed to prevent arrhythmias. One difficulty in answering this question is that determining the editing efficiency in vivo can be difficult. Whole myocardial tissue includes non-myocyte genomes that may not be as efficiently edited as cardiomyocytes with AAV9. Genome sequencing of RNA transcripts can give a more cardiomyocyte-specific readout, but nonsense-mediated decay from NHEJ will affect these readouts [47]. We have used in vivo AAV9 reporters as a proxy for genome editing, though it requires dual AAV transduction and can, therefore, underestimate the amount of editing [47]. Recent studies have used the ratio of mutant to wild-type alleles as a proxy for determining the efficiency of allele-specific genome editing [48]. Due to the lack of reliability in these methods, reported genome editing efficiencies necessary for preventing arrhythmias have ranged from 6 to 24% of the whole myocardium and less than 50% of myocytes.

One group has used computer modeling of ventricular tissues to determine the editing efficiency needed to prevent arrhythmogenesis [58]. One-, two-, and three-dimensional tissue models were used to simulate early and delayed afterdepolarization-triggered arrhythmias. Stabilizer cells were distributed to simulate cardiomyocyte gene therapy. Due to source–sink relationships in cardiac tissue, a minority of stabilizer cells (20–50%) was sufficient to prevent triggered activity. Clustering stabilizer cells reduced their efficacy, and higher-dimensional models required a greater percentage of gene-edited stabilizer cells. These modeling studies correlated with in vivo gene therapy studies but also reveal the importance of evenly distributed gene delivery. 

Even though CRISPR/Cas9 represents a promising approach, this genome editing system still has several limitations and associated risks, making it challenging to use in clinical trials. Delivery methods, editing efficiency, off-target effects, and immunogenicity are several major concerns that must be overcome. The most relevant limitations will each be discussed briefly in the following paragraphs:

*Delivery methods*: The CRISPR/Cas9 genome editing system requires the delivery of a poly II promoter with a gRNA sequence and a poly III promoter with a Cas9 protein in addition to a poly-A tail [59]. While there are many methods of delivering these genes to tissue, including injection of free plasmid, adenovirus, lentivirus, and nanoparticles, AAV vectors are most used for cardiac genome editing [60]. AAVs have the capability to transduce the entire myocardium effectively following intracoronary or intravenous injection without generating a large immune response and so have been the viral vector of choice for preclinical studies of cardiac gene editing. However, a limitation of AAVs is their packaging capacity which is limited to ~4.7–4.9 kb depending on the serotype. The most widely used Cas9 homolog, SpCas9 (from *Streptococcus pyogenes*), is 4.1 kb, and so requires the use of a minimal promoter or second AAV for the expression of gRNA. Other homologs such as SaCas9 (from *Staphylococcus aureus*) are smaller in size (3.1 kb), which provides the capacity to place gRNA and regulatory sequences into the same vector. Generally, the PAM sequence is dependent on which Cas9 homolog is used, and the smaller-sized homologs tend to have longer PAM sequences, which limits their targeting capability but increases their specificity.

Several groups have been able to expand the packaging capacity by using a second AAV vector with a split Cas9 protein. There are several methods to achieve this including overpacking, DNA homologous recombination, RNA splicing, and split-intein proteins [61]. Of the 13 main AAV serotypes, AAV8 and AAV9 have been used the most for post-natal cardiac genome editing, with AAV9 having the highest tropism to the heart [62]. Even though AAV9 has high tropism to the heart, it also has a great amount of tropism in the liver. As such, several publications focusing on de-targeting the liver have shown a greater cardiac specificity, though not necessarily a greater transduction efficiency [63]. Recently, evolution-directed in vivo screens of AAV9 capsids engineered to contain multimers have found success in finding muscle tropic AAV9s [64]. These are important for cardiomyopathies associated with dystrophic diseases such as DMD. Interestingly, these capsid variants showed an increased transduction efficiency in the myocardium in comparison with AAV9. One recombinant capsid, AAVrh74, is currently in clinical trial for gene therapy of micro-dystrophin [65]. Other recombinant capsids such as AAV-MYO-A1 have been used in vivo for genome editing of dystrophin with an eight-fold increase in vector delivery in the heart compared with AAV9 and a significant increase in corrected mRNA following genome editing of dystrophin [66].

*Editing efficiency and off-target effects*. At the date of this publication, three Cas9 homologs have been used to edit the genome of the heart for therapeutic effect: SaCas9 (1053 amino acids), SpCas9 (1368 amino acids), and *Campylobacter jejuni* Cas9 (CjCas9, 984 amino acids). Each Cas9 orthologue has a unique PAM that Cas recognizes to bind to DNA. Each homolog has a specific PAM sequence: SaCas9 (NNGRRT), SpCas9 (NGG), and CjCas9 (NNNNRYAC). The specificity of the PAM sequence restricts which DNA sequence can be cleaved, both on and off target. Additionally, work has been performed to modify existing Cas9 homologs to expand nuclease targeting capabilities. For example, the modified KKH SaCas9 (1053 amino acids) with variants in the DNA recognition domain has expanded PAM sequences of NNARRT, NNCRRT, and NNTRRT [67]. Screening of DNA-shuffled short Cas9 homologs called synthetic guided nucleases (1053–1054 amino acids) has identified a potentially more efficient and less restrictive nuclease with an NNGG PAM sequence. These improvements will allow the expanded targeting of Cas9 while still using a single AAV vector system to guide delivery.

The R221K and N394K mutations in SpCas9 have been shown to improve Cas9 nuclease activity by specifically enhancing the ability of SpCas9 to bind to DNA [68]. Furthermore, modifications of the nuclear localization sequences increase the activity of SpCas9 as well as precision editing systems. These and other regulatory elements can be used to enhance the efficiency of cardiac genome editing, provided there is capacity in the gene delivery vector. Another mutant form of Cas9, the D10A nickase, produces a single-strand nick at the target. Combining nickase Cas9 with a pair of gRNAs targeting complementary strands of the target gene greatly reduces off-target DSBs and improves specificity because this approach requires single-strand nicks at both sgRNA target sites to produce a DSB [69].

*Reducing immunogenicity*. Both *Staphylococcus aureus* and *Steptococcus pyogenes*, from which the most common Cas9 proteins (SaCas9 and SpCas9) are obtained, have infected humans for a long time [70]. The majority (58-78%) of humans had anti-Cas9 antibodies in their serum in one recent study [70]. Anti-Cas9 antibodies may lead to a fast degradation of the Cas9 proteins, preventing them from performing the desired genome-editing functions [71]. Strategies to overcome the limits posed by immunogenicity against Cas9 include gene editing treatment early in life and targeting immune-privileged organs. An immune privileged organ can be defined as a site where a graft tissue can be implanted without being rejected by the host due to an immunological reaction. Examples include the eyes, brain, placenta, fetus, and testicles [72]. For the treatment of cardiac arrhythmias, the latter approach could be exploited by performing prenatal or early postnatal delivery of the CRISPR/Cas9 system. For example, Nelson et al. [73] found that an immune response to AAV-CRISPR was minimized in post-natal day 2 neonatal mice. 

## 6. Conclusions and Future Directions

Cardiac genome editing with various CRISPR/Cas9-based technologies has emerged as a promising new therapeutic modality for inherited arrhythmia syndromes and cardiomyopathies. The CRISPR/Cas9 system has made the creation of cellular and animal models of cardiac arrhythmias more efficient and affordable. The feasibility of therapeutic genome editing using CRISPR/Cas9 has been demonstrated in various preclinical animal models. While innovations in technologies for cardiac gene delivery and targeting have resulted in improvements in editing efficacy in preclinical models, various challenges remain related to delivery methods, editing efficiency, off-target effects, and immunogenicity. The first clinical trials for inherited conditions associated with cardiomyopathies are being initiated, and it is likely that similar studies will commence soon for arrhythmogenic cardiomyopathy or inherited arrhythmia syndromes. The rate of progress in cardiac genome editing has been remarkable given that the discovery of CRISPR/Cas9 as a molecular tool occurred only a little over a decade ago. We can look forward to tremendous progress in genome editing approaches and likely clinical applications in the next decade.

## Figures and Tables

**Table 1:** Therapeutic gene editing studies in mouse models of inherited arrhythmias.

Gene	Disease	Cas9	Vector	Route	Age	Dose/Mouse	Editing Efficiency *	Physiological Effect	Reference
*PRKAG2*	PRKAG Syndrome	SpCas9	AAV9	IV	1 wks	5 × 10^11^ vg	2.6–6.5%	Reduced stress-induced VT	[46]
*RYR2*	CPVT	SaCas9	AAV9	SQ	1 wks	1 × 10^12^ vg	11%	Prevented stress-induced VT	[47]
*DMD*	Duchenne’s Muscular Dystrophy	ABE8e/ Prime Editor	Dual AAV9	TC	2 wks	1.25 × 10^12^ vg	6.7–35.0%	Normalized arrhythmic calcium traces of IPSC-cardiomyocytes	[42]
*PLN*	DCM/ARVC	SaCas9	AAV9	TV	8 wks	4.5 × 10^12^ vg	7%	Reduced stress-induced VT	[48]

Legend: ABE8e, adenosine base editor 8; ARVC, arrhythmogenic right ventricular cardiomyopathy; CPVT, catecholaminergic polymorphic ventricular tachycardia; DCM, dilated cardiomyopathy; DMD, dystrophin; IV, intravenous; PLN, phospholamban; PRKAG2, 5′-AMP-activated protein kinase subunit gamma-2; RYR2, ryanodine receptor type 2; SaCas9, *Staphylococcus aureus* Cas9; SpCas9, *Streptococcus pyogenes* Cas9; SQ, subcutaneous; TC, thoracic cavity injection; TV, tail vein injection; vg, vector genomes; VT, ventricular tachycardia; * based on NGS on-target editing.

## Data Availability

All the data are available in the text, tables, and figures of the review.

## Abbreviations

Cas	CRISPR-associated protein
CPVT	catecholaminergic polymorphic ventricular tachycardia
CRISPR	clustered regularly interspaced short palindromic repeats
dCas	endonuclease deficient CRISPR-associated protein
DMD	Duchenne’s muscular dystrophy
DSB	double-stranded DNA break
gRNA	guide RNA
HDR	homology-directed repair
iPSC	induced pluripotent stem cell
LQTS	long QT syndrome
nCas9	nicking CRISPR-associated
NHEJ	non-homologous end joining
PAM	protospacer adjacent motif
